# Prevalence of Long-COVID Among Low-Income and Marginalized Groups: Evidence From Israel

**DOI:** 10.3389/ijph.2022.1605086

**Published:** 2022-11-28

**Authors:** Oren Heller, Yung Chun, Stav Shapira, Aron Troen, Yaniv Shlomo, Mary Acri, Phillip Marotta, Saneel Kulkarni, Brendan Kinnison, Michal Grinstein-Weiss

**Affiliations:** ^1^ Washington University in St. Louis—Social Policy Institute (SPI), St. Louis, MO, United States; ^2^ School of Public Health, Faculty of Health Sciences, Ben-Gurion University of the Negev, Be’er-sheva, Israel; ^3^ The School of Nutrition Science, The Institute of Biochemistry Food Science and Nutrition, The Robert H. Smith Faculty of Agriculture Food and Environment, The Hebrew University of Jerusalem, Rehovot, Israel; ^4^ Washington University in St. Louis—George Warren Brown School of Social Work, St. Louis, MO, United States; ^5^ Centene Corporation, St. Louis, MO, United States

**Keywords:** health inequalites, SARS-CoV-2, Israel, long-COVID, low-income, marginalized groups

## Abstract

**Objective:** To identify the socioeconomic and demographic factors associated with the prevalence of self-reported long-COVID symptoms.

**Method:** We examined the association between acute-COVID (SARS-CoV-2) and long-COVID symptoms, by a cross-sectional analysis of data obtained on a prospective online-survey, conducted from November to December 2021 on a nationally-representative sample of the Israeli population (N = 2,246).

**Results:** Findings suggest that there is a greater likelihood of experiencing long-COVID symptoms among low-income and among marginalized groups. After controlling for demographic and socioeconomic attributes, those who had moderate/severe acute-COVID were 1.3 (*p* < 0.05) times more likely to experience a long-term symptom and also reported more long-term symptoms (2.2 symptoms) than those who have not been infected (1.4 symptoms; *p* < 0.01). Among the low-income group, a larger gap in symptom count was found between those who had moderate/severe acute-COVID (3.3 symptoms) and those who had not been infected (1.8 symptoms, *p* < 0.05).

**Conclusion:** Our findings highlight the importance of raising awareness of long-COVID among marginalized population groups, and to the therapeutic options available. Such efforts should be tailored and should consider the unique socioeconomic and cultural characteristics, as well as the preexisting low access to healthcare services among these groups.

## Introduction

Since the beginning of the coronavirus pandemic, approximately 435 million individuals have been infected worldwide and 5.9 million people have died from COVID-19 related illnesses (1, 2). At the time of writing, in Israel, approximately 3.6 million people out of a total population of 9.3 million have been infected and over 10,000 people have died of the disease (3). Although COVID-19 was initially considered to be primarily a respiratory disease, it is now evident that the virus may harm the pulmonary, cardiovascular, neurologic, psychiatric, gastrointestinal, renal, endocrine, and musculoskeletal systems, in both adult and pediatric populations (4, 5), and more rarely, in children, can lead to a life threatening multisystem inflammatory syndrome (MIS-C) (6). While the acute symptoms of COVID-19 infection typically remit after several weeks, a concerning proportion of individuals experience lingering symptoms that persist for months after infection (1, 7). Symptoms that appear during or after COVID-19 onset, and which persist for one month or more, are referred variably in the literature as post-COVID, post-COVID syndrome, post-acute COVID, long-haul COVID, or simply, “long-COVID”. There is considerable variability with respect to the presentation of long-COVID: some individuals experience a continuation of the same symptoms as when they were first infected; others may experience an asymptomatic period followed by a recurrence of symptoms; and still others present with symptoms that are different from when they were first infected (7). Symptoms and conditions most commonly associated with long-COVID include shortness of breath and fatigue and may also include dizziness, chest and abdominal pain, and mental health problems such as depression, anxiety, and posttraumatic stress disorder (5, 8–12).

Long-COVID is difficult to diagnose and treat, not only because the disease is still an evolving phenomenon but also as a result of its varied presentation, lack of specificity, and temporal separation from the acute infection. Nevertheless, it can significantly degrade quality of life and wellbeing (13, 14). Moreover, there is growing evidence that long-COVID may be related to underlying damage to major organs including the lungs, heart and cardiovascular system, liver, kidneys, and brain (1). Recently, large population studies have shown that individuals who recovered from COVID-19 have significantly increased risk of incident cardiovascular disease (15), mental health disorders (15, 16), and brain anomalies on MRI (17) even among confirmed cases whose acute infection was mild or asymptomatic. Thus, with millions affected worldwide, and as the world transitions to “living with COVID” (18), understanding the nature and determinants of long-COVID is an increasingly urgent public health concern (19–22).

As we have already learned with respect to acute-COVID, social and economic disparities are among the most important predictors of disease severity and outcome and of willingness to accept public health guidance, including on vaccination. It stands to reason that these factors will also be important in determining the outcomes of long-COVID because of the close relationship between the incidence and severity of the acute disease, with disparities in education and income, food insecurity and obesity, as well as vaccine hesitancy among ethnic minorities and other marginalized populations (22–30). However, little is known about the social and economic determinants of long-COVID. Clarifying their role in this chronic and prevalent condition will be necessary if we are to improve clinical care for this condition and develop effective and equitable public health policies to deal with its consequences.

Although still in infancy, a growing set of studies have examined risk factors associated with long-COVID. Older adults and women have been found to be at a heightened risk for onset (31, 32) as have persons with high body mass and those who had reported more than five symptoms when initially infected (31). Co-morbid health (asthma, obesity) and mental health problems prior to the pandemic have also been linked to the onset of long-COVID (32). In light of these studies, we aimed to identify the socioeconomic and demographic factors associated with the prevalence of self-reported long-COVID symptoms using cross-sectional data obtained from our prospective online survey of a nationally-representative sample of the Israeli population.

## Methods

### Study Design and Population

This study utilized data obtained by a longitudinal online Qualtrics survey of a nationally-representative sample of Israeli adults, which was repeated five times during the COVID-19 pandemic, between June 2020 to November 2021. The objective of the parent study was to evaluate how socioeconomic factors affect resilience during a pandemic for the purpose of policy research and evaluation. For this analysis, we used data from the 5th round of the survey (*n* = 2,362), which was conducted between 26 November and 21 December 2021. This corresponded to the period in Israel between the fourth wave, dominated by the Delta variant, and the fifth “omicron” wave, which began in early 2022*.* The sample was constructed from a pool of participants by a local survey company using quota sampling techniques to ensure that the sample represented Israel demographic characteristics with respect to gender, age groups, income groups, and religion/religiosity. The 5th round sample was constructed from returning participants (*n* = 1,644; 69.6%) whose participation rate was 45.0%, and new participants (*n* = 718; 30.4%) whose participation rate was 6.4%. We excluded participants who had acute-COVID less than one month before the survey (*n* = 35) and participants who did not respond to key items, using listwise deletion. Since sample composition was the same as in the parent survey with respect to demographic characteristics and cases of acute-COVID (Table 1), we assumed that the exclusion of records was random. The final analytical sample included all participants with complete responses (*n* = 2,246).

**TABLE 1:** Sample composition (Israel, 2021).

	Parent Sample	Study Sample
Total	2362 (100%)	2246 (100%)
Gender		
Male	1079 (45.7%)	1023 (45.6%)
Female	1278 (54.1%)	1223 (54.5%)
Other	4 (0.2%)	
Missing	1 (0.0%)	
Age group		
18–29	508 (21.5%)	466 (20.8%)
30–39	566 (24.0%)	535 (23.8%)
40–54	580 (24.6%)	554 (24.7%)
55+	708 (30.0%)	691 (30.8%)
Ethnicity/religiosity		
Non-Ultra-Orthodox Jew	1840 (77.9%)	1787 (79.6%)
Ultra-Orthodox Jew	195 (8.3%)	190 (8.5%)
Arab Israeli	279 (11.8%)	241 (10.7%)
Other	31 (1.3%)	28 (1.3%)
Missing	17 (0.7%)	
Number of children		
None	1266 (53.6%)	1228 (54.7%)
1–2 children	721 (30.5%)	684 (30.5%)
3+ children	343 (14.5%)	332 (14.8%)
Missing	32 (1.4%)	2 (0.1%)
Number of adults		
1 adult	296 (12.5%)	284 (12.6%)
2 adults	1243 (52.6%)	1205 (53.7%)
3+ adults	782 (33.1%)	742 (33.0%)
Missing	41 (1.7%)	15 (0.7%)
Average household monthly income		
Low-income	436 (18.5%)	401 (17.9%)
Middle-income	570 (24.1%)	565 (25.2%)
High-income	1081 (45.8%)	1060 (47.2%)
Missing	275 (11.6%)	220 (9.8%)
COVID-19 infection and short-term symptoms		
Not infected	2012 (85.2%)	2004 (89.2%)
Infected and had mild/minor symptoms	185 (7.8%)	159 (7.1%)
Infected and had moderate/severe symptoms	93 (3.9%)	83 (3.7%)
Missing	72 (3.1%)	
By average household monthly income		
Low-income (100%)		
Not infected	353 (81.0%)	338 (84.3%)
Infected and had mild/minor symptoms	48 (11.0%)	41 (10.2%)
Infected and had moderate/severe symptoms	25 (5.7%)	22 (5.5%)
Middle-income (100%)		
Not infected	481 (84.4%)	490 (86.7%)
Infected and had mild/minor symptoms	54 (9.5%)	50 (8.9%)
Infected and had moderate/severe symptoms	33 (5.8%)	25 (4.4%)
High-income (100%)		
Not infected	979 (90.6%)	979 (92.4%)
Infected and had mild/minor symptoms	67 (6.2%)	54 (5.1%)
Infected and had moderate/severe symptoms	25 (2.3%)	27 (2.6%)
By ethnicity/religiosity		
Non-Ultra-Orthodox Jew (100%)		
Not infected	1650 (89.7%)	1648 (92.2%)
Infected and had mild/minor symptoms	107 (5.8%)	91 (5.1%)
Infected and had moderate/severe symptoms	51 (2.8%)	48 (2.7%)
Ultra-Orthodox Jew (100%)		
Not infected	118 (60.5%)	118 (62.1%)
Infected and had mild/minor symptoms	56 (28.7%)	54 (28.4%)
Infected and had moderate/severe symptoms	19 (9.7%)	18 (9.5%)
Arab Israeli (100%)		
Not infected	212 (76.0%)	211 (87.6%)
Infected and had mild/minor symptoms	20 (7.2%)	13 (5.4%)
Infected and had moderate/severe symptoms	22 (7.9%)	17 (7.1%)
Time passed since last positive COVID-19 test		
Less than 1 month	35 (1.5%)	
1 to 6 months	85 (3.6%)	85 (3.8%)
6 months or more	151 (6.4%)	150 (6.7%)
Missing	7 (0.3%)	
Not infected	2084 (88.2%)	2011 (89.5%)
COVID-19 vaccination		
Not vaccinated	209 (8.9%)	190 (8.5%)
Received 1 dose	100 (4.2%)	91 (4.1%)
Received 2 doses	331 (14.0%)	313 (13.9%)
Received 3 doses	1657 (70.2%)	1624 (72.3%)
Missing	65 (2.8%)	28 (1.3%)

The survey inquired respondents’ demographic and socioeconomic characteristics and included COVID-19 related questions: having past diagnosis of acute COVID, disease severity, time passed since the diagnosis, and vaccination for COVID-19 including the number of shots received and the time passed since the last one. The survey also inquired whether and how long respondents, regardless of their past infection with COVID-19, currently are suffering from a range of symptoms, including fatigue, shortness of breath, impaired memory and attention, continuous damage to the sense of taste and the sense of smell, muscle aches and joint pains, chest pain, headaches, accelerated heartbeat, hair loss, sore throat and earache, and dizziness (33). Using these reports, we constructed a variable indicating presence of at least one symptom and a variable counting the number of symptoms one suffers from.

For the purpose of this analysis, we considered the frequency of symptoms among uninfected individuals as the background prevalence of these conditions and the prevalence of persistent symptoms among those diagnosed with acute COVID as evidence of long-COVID.

### Empirical Model Design

We explored the association of the prevalence of long-COVID with the diagnosis and severity of acute COVID-19 in a cross-sectional design, using three types of models: 1) logistic regression models predicting presence of at least one of the relevant self-reported symptoms; 2) linear models predicting the number of self-reported symptoms, ranging from 0 to 11; and 3) for each symptom s of the self-reported symptoms, a logistic regression model predicting its prevalence.

For models 1 and 3, the following logistic model is employed:

$$\log\frac{\mathrm{Pr}\left(Y_{s}=1\right)\;}{\left(1-\mathrm{Pr}\left(Y_{s}=1\right)\;\right)}=\beta_{s}+X^{demo}\beta_{s}^{demo}+X^{SE}\beta_{s}^{SE}+X^{COVID}\gamma_{s}+X^{VAC}\beta_{s}^{VAC}+\varepsilon_{s}$$

For model 2, the following linear model is employed:

$$\sum Y_{s}=\beta_{OLS}+X^{demo}\beta_{OLS}^{demo}+X^{SE}\beta_{OLS}^{SE}+X^{COVID}\gamma_{OLS}+X^{VAC}\beta_{OLS}^{VAC}+\varepsilon_{OLS}$$

where $Y_{s}$ is a binary variable indicating having the symptom; $Y_{s}=1$ if one would report having at least one of the 11 symptoms (model 1) or having symptom s (models 2 and 3), otherwise $Y_{s}=0$ . $X^{demo}$ includes a set of demographic characteristics of survey respondents, including gender, age (18–29, 30–39, 40–54, and 55 or above), and ethnicity/religiosity (general Jews—hereafter non-ultra-orthodox Jew, ultra-orthodox Jews, and Arab Israelis). $X^{SE}$ refers to socio-economic status captured by household income-per-capita (household income divided by household size) relative to its median value (NIS 4,167) in the study sample (low-income for less than half the median, middle-income for half the median to median, and high-income for higher than median). $X^{COVID}$ refers to a past infection of COVID-19 and disease severity (not infected, infected and had mild/minor symptoms, infected and had moderate/severe symptoms). $X^{VAC}$ refers to the COVID-19 vaccine, including the number of shots received (none, 1, 2 or 3) and the time passed since the last shot (up to a week, 1–2 weeks, 2 weeks to 1 month, 1–2 months, 2–3 months, 3–6 months, 6 months or more). To examine whether long-COVID symptom prevalence among those who were infected varies with individuals’ characteristics, we also estimated the above models with an interaction term of $X^{COVID}$ with household income, age, and ethnicity/religiosity.

For a better representation of the regression results, we estimated and plotted the predicted margins—predicted probability (logistic regressions) and predicted count (linear regressions) of long-COVID symptoms—by COVID-19 infection and severity, by income, and by religiosity (see also Supplementary Appendix Table SA1). The data analysis in this study was conducted using Stata (Version 16; StataCorp, 2019), and we used a threshold of *p* < 0.10 to assess the statistical significance.

## Results

### Study Sample

Study sample comprises 2,246 individuals of which 89.2% have not had COVID-19, 7.1% have had COVID-19 with mild/minor symptoms, and 3.7% have had COVID-19 with moderate/severe symptoms.

### Long-Term COVID-Related Symptoms

**FIGURE 1:**
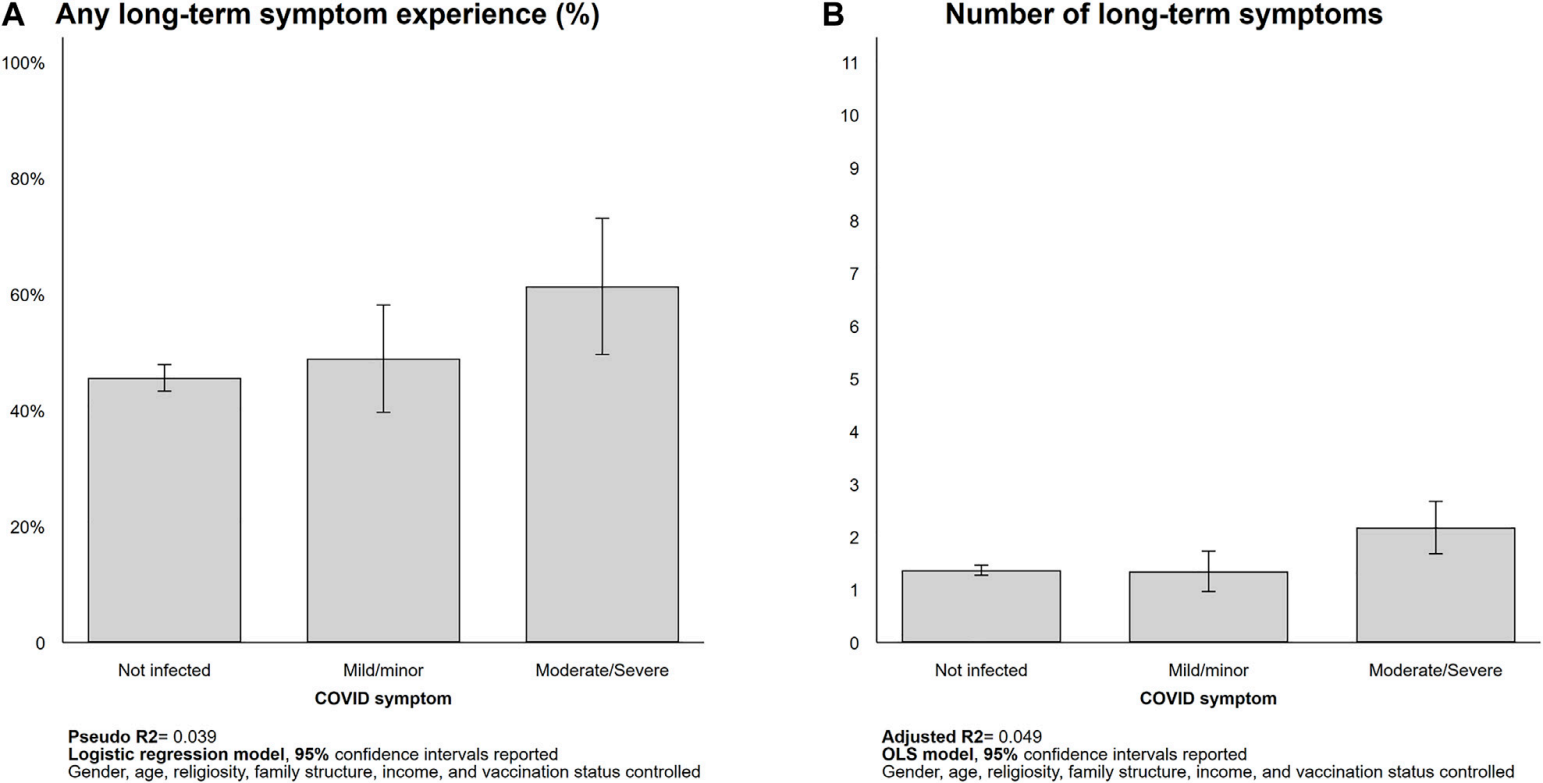
Long-term symptom prevalence (Israel, 2021). Predicted probability [**(A)**, logistic regression] and counts of long-term symptom [**(B)**, OLS] reported.

Next, we report the results from our multivariate regression models showing how the self-reported COVID-19 long-term symptom (hereafter long-term symptom) prevalence varied with COVID-19 infection and severity of short-term symptoms. On average, 46.7% of our respondents reported one or more long-term symptoms. Also, we found that the long-term symptom prevalence varied with COVID-19 infection as well as its severity. Even after controlling for demographic and socioeconomic attributes, those who suffered from moderate or severe COVID-19 short-term symptoms were 1.3 times more likely to experience a long-term symptom than those who had not been infected (Not infected = 45.6%; Moderate/severe = 61.4%; *p* < 0.05; Figure 1A). Furthermore, those with moderate/severe COVID-19 reported more long-term symptoms on average (2.2) than those with mild/minor symptoms (1.3, not significant) and those who did not get infected (1.4, *p* < 0.01; Figure 1B).

**FIGURE 2:**
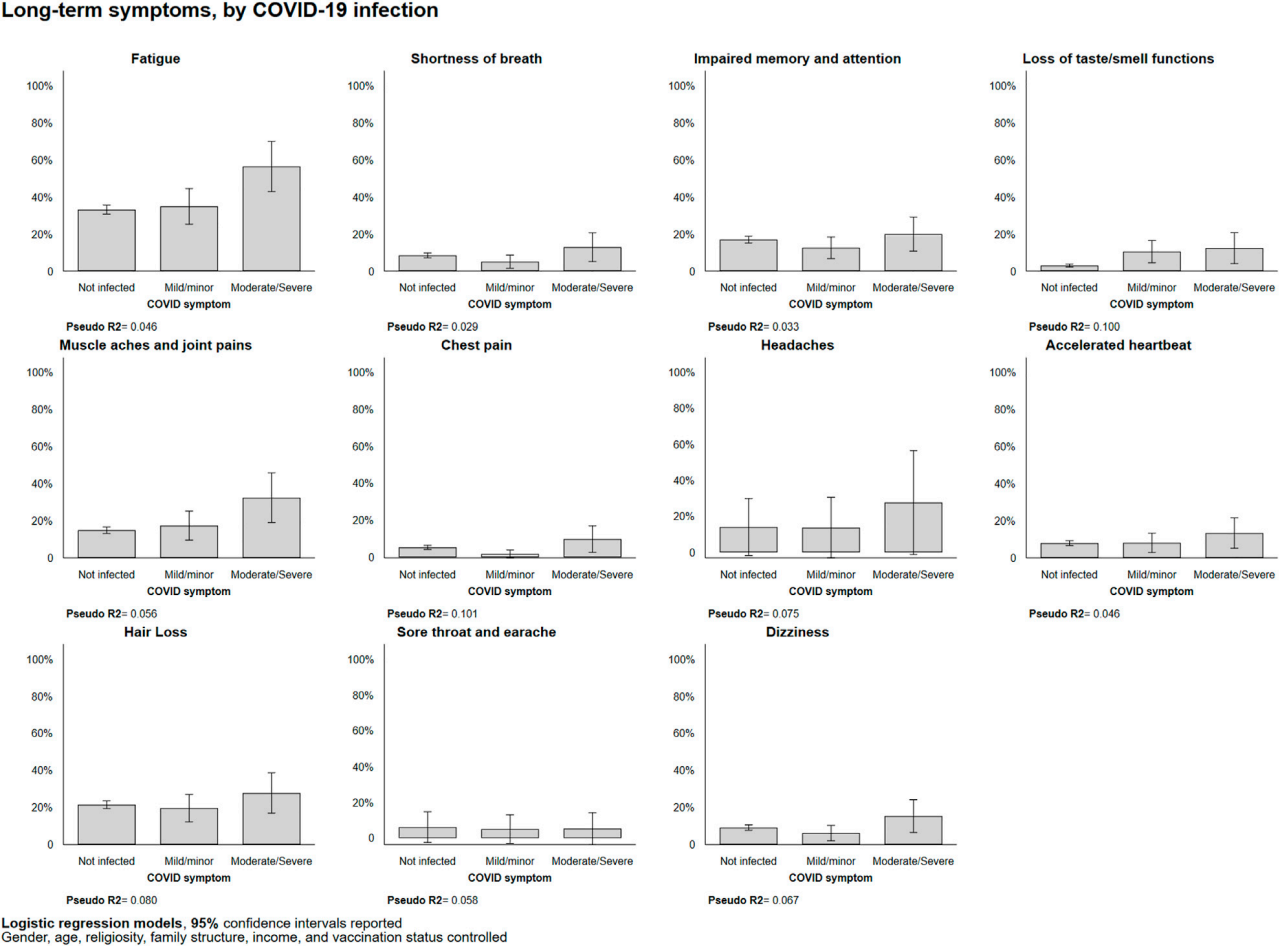
Itemized long-term symptom experiences by COVID-19 infection, marginal probabilities (Israel, 2021). Predicted probabilities from logistic regressions reported.

For some symptoms, we observed a greater gap between the moderate/severe group and the mild/minor group than between the mild/minor and not-infected groups: fatigue (not infected = 33.2%; moderate/severe = 56.4%; *p* < 0.01) and muscle aches and joint pains (not infected = 14.9%; moderate/severe = 32.4%; *p* < 0.05). Furthermore, in the case of loss of taste/smell functions, regardless of the severity of COVID-19 symptoms, those who had been infected (mild/minor = 10.5%; moderate/severe = 12.4%) were 3.5 and 4.1 times more likely to report this symptom than those who had not been infected (3.0%; *p* < 0.05 for both comparisons). For other long-COVID symptoms, such as shortness of breath, muscle aches and joint pains, and headaches, we observed similar, though statistically not significant, relationships with acute-COVID disease and severity (Figure 2).

### Long-Term COVID-Related Symptoms and Individual Characteristics

Lastly, we utilized the same sets of multivariate regression models to explore how the associations between long-term COVID-19 symptom prevalence and short-term COVID-19 infection/severity varied across respondents’ socioeconomic characteristics (i.e., income and religiosity).

#### Long-Term Symptoms by Income

**FIGURE 3:**
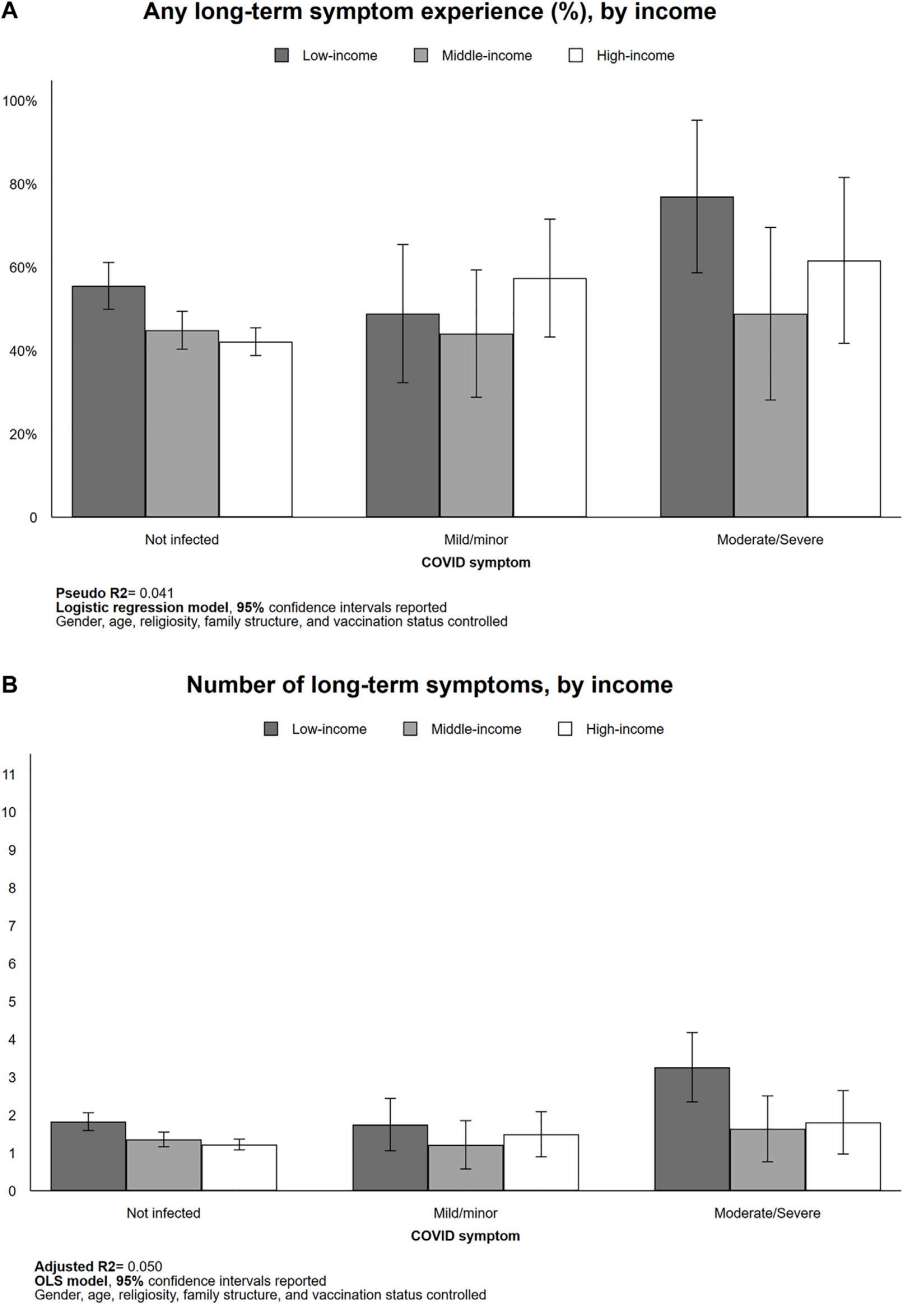
Long-term symptom experiences by income (Israel, 2021). Predicted probability [**(A)**, logistic regression] and counts of long-term symptom [**(B)**, OLS] reported.

We observed a negative association between income and long-term symptom experience in those without COVID-19 infection experience, both in experiencing at least one long-COVID symptom (low-income = 55.5%; high-income = 42.1%; *p* < 0.01) and in symptom count (low-income = 1.8; high-income = 1.2; *p* < 0.01). We also observed higher rates of long-term symptom prevalence in both low- and high-income respondents who had moderate/severe COVID-19 symptoms relative to those without COVID-19 infection (low-income = 55.5%–77.0%, *p* < 0.10; high-income = 42.2%–61.7%, *p* < 0.10; See Figure 3A). Also, the count of long-term symptoms increased by 1.8 in the low-income group (1.8–3.3, *p* < 0.05) but not in the other two groups. That is, the count of long-term symptoms of respondents who had moderate/severe COVID-19 symptoms was the highest among low-income respondents (3.3 compared to middle-income = 1.6, *p* < 0.10; and high-income = 1.8, not significant; Figure 3B).

In the low-income cohorts, we observed a statistically significant higher rate of long-term fatigue (46.6%–58.0%, *p* < 0.05), muscle aches and joint pains (19.9%–64.1%, *p* < 0.01). In the high-income group, we observed a statistically significantly higher rate of fatigue (29.0%–58.0%, *p* < 0.05) and loss of taste/smell functions (1.8%–26.0%, *p* < 0.05; See Supplementary Appendix Figure SA1).

#### Long-Term Symptoms by Ethnicity/Religiosity

**FIGURE 4:**
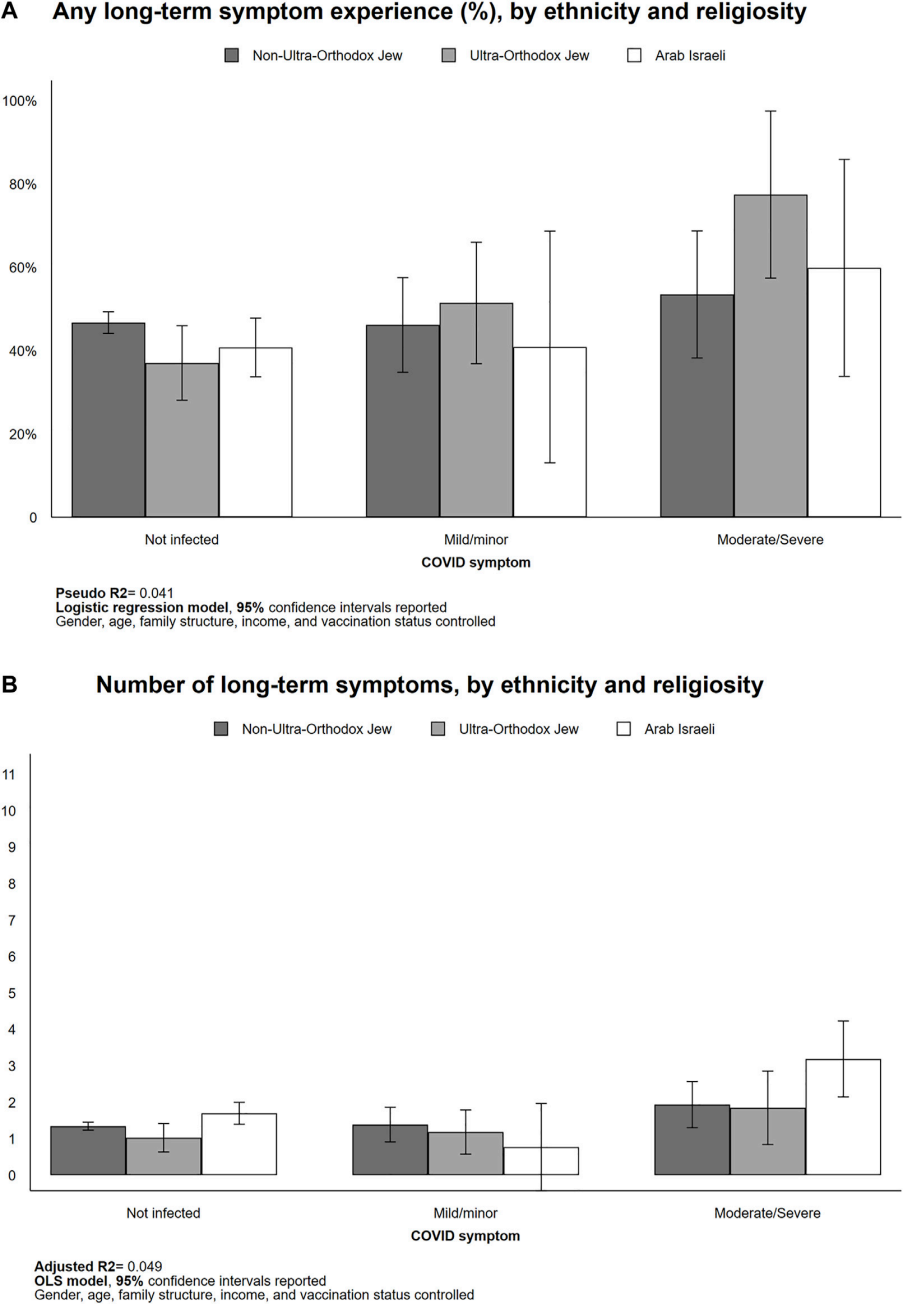
Long-term symptom experiences by ethnicity/religiosity (Israel, 2021). Predicted probability [**(A)**, logistic regression] and counts of long-term symptom [**(B)**, OLS] reported.

Each of the three examined population groups comprising the Israeli society experienced more long-term symptoms if they had had moderate/severe COVID-19 disease, relative to those who were uninfected. Nevertheless, there were substantially higher, but not necessarily statistically significant, rates of long-term symptoms among non-Ultra-Orthodox Jews (46.9%–53.3%, not significant), the Ultra-Orthodox Jews (37.5%–76.1%, *p* < 0.05), and the Arab Israelis (41.2%–58.6%, not significant; see Figure 4A). On the other hand, Arab Israelis exhibited a significantly higher number of long-term symptoms as they suffered moderate/severe COVID-19 symptom in the short-term (1.7–3.2, *p* < 0.05). Among those who have not been infected with COVID-19, the Arabs Israelis had more long-COVID symptoms than the other group (1.7 symptoms relative to 1.3 (non-Ultra-Orthodox Jew, *p* < 0.10) and 1.1 (Ultra-Orthodox Jew, *p* < 0.10); Figure 4B).

Non-Ultra-Orthodox Jews exhibited significantly higher rates of certain long-term symptoms, including fatigue (33.9%–53.9%, *p* < 0.10), loss of taste/smell functions (2.6%–19.3%, *p* < 0.05), and muscle and joint pains (14.5%–30.6%, *p* < 0.10) as they experienced moderate/severe short-term COVID-19 (See Supplementary Appendix Figure SA2). Note that the statistically non-significant differences in short-term COVID-19 severity for the two minority groups (Ultra-Orthodox Jews and Arab Israelis) might be related to their small sample sizes (Ultra-Orthodox Jews = 190; Arab Israelis = 241).

## Discussion

This study explored the reported prevalence of COVID-19 long-term symptoms and how these symptoms vary with infection and severity of acute-COVID and by demographic and socioeconomic characteristics. The findings indicated that those infected with COVID-19 and who suffered moderate to severe illness were more likely to both experience long-COVID and to suffer more symptoms than those who experienced minor illness or were not infected at all. These findings align with those of a systematic review that found the severity of initial infection or symptom load is associated with a greater likelihood of long-COVID symptomatology (34), as well as with those of a recent German study which reported the intensity of acute phase symptoms as a predictor of post-COVID syndrome (35). Thus, our results further stress the importance of developing proactive and standardized practices for monitoring post-infection symptomatology to combat long-COVID (36, 37). Furthermore, we found that belonging to low-income and/or to a marginalized social or ethnic group was associated with an even greater likelihood of experiencing long-COVID. Our results are consistent with previous findings identifying specific subgroups as more vulnerable to both acute-COVID (38) and several symptoms of long-COVID (39–41). Additionally, recent evidence indicates higher rates of healthcare utilization (e.g., visits with a primary care provider) in the year following the acute COVID infection among socially vulnerable populations (42), highlighting the potential burden on healthcare facilities and staff among marginalized communities. Notably, in the current analysis, individuals from the high-income group that experienced mild or severe acute-COVID symptomatology also reported relatively high rates of long-term symptoms. This finding may reflect the documented association between income levels and COVID awareness (43) and suggest that the mechanisms involved in experiencing long-COVID are complex and warrant further research. Further discussion of these findings, their clinical and social implications, and future research directions are elaborated on below.

The findings relating to specific population groups that were more susceptible to long-COVID raise interesting health and social issues. Individuals of marginalized groups, whether due to ethnicity, social affiliation, low income, or other reasons, are known to be highly vulnerable in emergencies and to be disproportionately affected by them (44). The increased vulnerability stems from various barriers which impede the access of these groups to a variety of resources such as healthcare (45) and education (46), consequently leading to inequalities and blocking life opportunities (47). The COVID-19 pandemic is a stark and ongoing example of the complex ways in which marginalization materializes in adverse outcomes. In Israel, acute-COVID infections and mortality were higher in communities with predominantly Arab and ultra-Orthodox Jewish populations, which also tend to have lower income rates than the general Jewish population (48). Conversely, vaccination was lower among the same populations, despite access to universal healthcare and the rapid rollout of COVID-19 vaccination in Israel (49). The increased vulnerability of these groups has been attributed to various factors such as crowded living conditions, increased dependency on public transportation, employment in occupations that do not allow for the maintenance of social distancing, and a lack of trust in state institutions and their guidelines (50). Our findings provide the first insights into the association between COVID long-term risks and ethnicity, religiosity, and income levels in Israel. The results highlight ultra-Orthodox and Arab Israelis as also highly vulnerable to long-COVID. However, the low vaccine uptake reported above cannot explain these results as we have controlled for vaccination status in our models. The current findings emphasize the importance of raising awareness of long-COVID among these population groups and of the therapeutic options available to those in need. Awareness efforts should be tailored to these groups and should consider their unique socioeconomic and cultural characteristics as well as their preexisting low access to healthcare services (50). For example, several studies suggest using remote home monitoring to manage post-COVID recovery and rehabilitation using telemedicine platforms and devices (51, 52). However, it should be noted that the rate of telemedicine use among marginalized groups is relatively low (53, 54). Thus, close attention should be paid to ensure that the use of telemedicine in this context does not exacerbate existing inequalities.

The challenges of studying long-COVID are considerable (55). It is difficult to attribute the presence of prevalent, persistent, and diffuse or non-specific symptoms to the initial infection with certainty, particularly in cross-sectional and retrospective studies that are susceptible to recall bias. It is important to obtain a diagnosis of the initial infection and to demonstrate that the post-acute symptoms are sequalae of the infection that did not pre-date it. Thus, prospective studies are needed to identify risk factors for incident long-COVID by comparing exposures and outcomes between cases of long-COVID, acute only cases, and healthy controls. The inclusion of questions pertaining to long-COVID in the ongoing phase of our study will allow us to study incident long-COVID in future waves.

The current study has several limitations. First, the study relies on cross-sectional and self-reported data, which may lead to bias and inaccuracy in estimating the phenomenon under investigation, namely long-COVID. Moreover, it is reasonable to assume that a certain proportion of the participants experienced asymptomatic acute-COVID and were not officially diagnosed (56), which may cause additional bias. However, even if this figure turns out to be accurate, it will strengthen the research findings and not weaken them. Second, the issue of sample selection may also be the cause of potential bias: the rate of participants from Arab Israeli households in the current sample (11%) is slightly lower than their actual rate (15%) (57). However, here as well, our findings suggest that increasing their rate will strengthen the findings rather than weaken them. Finally, the current analysis evaluated the direct effects of marginalization indicators (e.g., belonging to a low-income household, ethnicity, etc.) on long-COVID symptom prevalence but did not measure their potential indirect effect. This important issue remains the goal of future research.

Despite these limitations, the results presented here help broaden our understanding of the relatively understudied phenomenon of long-COVID by offering insights into the attributes and predictors of this phenomenon, as well as specific populations that are at risk of experiencing it. As the pandemic continues for the foreseeable future and as the current surge of morbidity has led to more than 27 million new or recurrent acute-COVID cases in the US alone (7), it is of utmost importance to prepare for the aftermath of the infection. The current findings can serve as the basis for future healthcare planning and as a launchpad for future investigations of researchers and public health officials worldwide into how to better cope with the long-term consequences of the pandemic. Finally, as long-COVID is still a phenomenon that needs to be unraveled, further research is needed to better characterize the disease and its effects and to explore effective treatment and rehabilitative interventions.
