# Supplementary material for: Prevalence of Long-COVID Among Low-Income and Marginalized Groups: Evidence From Israel
Source: Int J Public Health. 2022 Nov 28;67:1605086. doi: 10.3389/ijph.2022.1605086 (PMC9742204; doi:10.3389/ijph.2022.1605086)
Supplement: Supplementary file 1 [file Table1.docx]

**Appendix Table A1. Regression estimation results predicting probability and count of long-COVID symptoms (Israel, 2021)**

|  | Logistic regression estimated odds ratios (Dependent variable=having at least one long-COVID symptom) | | | Linear regression estimated coefficients (Dependent variable=long-COVID symptom count) | | |
| --- | --- | --- | --- | --- | --- | --- |
|  | Long-term symptom prevalence by COVID-19 infection & short-term symptoms | Long-term symptom experiences by income | Long-term symptom experiences by ethnicity / religiosity | Long-term symptom prevalence by COVID-19 infection & short-term symptoms | Long-term symptom experiences by income | Long-term symptom experiences by ethnicity / religiosity |
|  | (1) | (2) | (3) | (4) | (5) | (6) |
| **Gender (ref. Male)** |  |  |  |  |  |  |
| Female | 2.158*** | 2.145*** | 2.179*** | 0.716*** | 0.717*** | 0.719*** |
|  | (0.191) | (0.191) | (0.194) | (0.092) | (0.092) | (0.092) |
| **Age Group (ref. 18-29)** |  |  |  |  |  |  |
| 30-39 | 0.997 | 0.997 | 0.993 | 0.075 | 0.066 | 0.077 |
|  | (0.132) | (0.133) | (0.132) | (0.137) | (0.138) | (0.138) |
| 40-54 | 1.079 | 1.082 | 1.071 | 0.150 | 0.147 | 0.148 |
|  | (0.144) | (0.145) | (0.143) | (0.138) | (0.138) | (0.138) |
| 55+ | 1.112 | 1.120 | 1.110 | 0.191 | 0.187 | 0.194 |
|  | (0.150) | (0.152) | (0.150) | (0.140) | (0.140) | (0.140) |
| **Ethnicity/religiosity (ref. Non-Ultra-Orthodox Jew)** |  |  |  |  |  |  |
| Ultra-Orthodox Jew | 0.859 | 0.912 | 0.671** | -0.265 | -0.230 | -0.317 |
|  | (0.144) | (0.155) | (0.137) | (0.174) | (0.176) | (0.207) |
| Arab Israeli | 0.803 | 0.798 | 0.785 | 0.352** | 0.341** | 0.352** |
|  | (0.122) | (0.122) | (0.126) | (0.156) | (0.157) | (0.166) |
| Other | 1.994* | 1.979* | 1.857 | 0.998** | 0.984** | 1.006** |
|  | (0.810) | (0.806) | (0.763) | (0.409) | (0.409) | (0.416) |
| **Average household monthly income (ref. Low-income)** |  |  |  |  |  |  |
| Middle-income | 0.647*** | 0.652*** | 0.639*** | -0.530*** | -0.468*** | -0.522*** |
|  | (0.089) | (0.097) | (0.088) | (0.142) | (0.154) | (0.143) |
| High-income | 0.615*** | 0.583*** | 0.615*** | -0.625*** | -0.603*** | -0.615*** |
|  | (0.080) | (0.081) | (0.080) | (0.135) | (0.144) | (0.135) |
| **COVID-19 infection & short-term symptoms (ref. Not infected)** |  |  |  |  |  |  |
| Infected and had silent/light symptoms | 1.143 |  |  | -0.022 |  |  |
|  | (0.229) |  |  | (0.208) |  |  |
| Infected and had moderate/severe symptoms | 1.896** |  |  | 0.808*** |  |  |
|  | (0.495) |  |  | (0.263) |  |  |
| **COVID-19 vaccination (ref. Not vaccinated)** |  |  |  |  |  |  |
| Received 1 dose | 4.475* | 4.662* | 4.236 | 0.683 | 0.748 | 0.620 |
|  | (3.956) | (4.132) | (3.753) | (0.819) | (0.820) | (0.820) |
| Received 2 dose | 4.002* | 4.147* | 3.778 | 0.647 | 0.666 | 0.607 |
|  | (3.372) | (3.499) | (3.189) | (0.772) | (0.772) | (0.772) |
| Received 3 doses | 3.562 | 3.714 | 3.338 | 0.631 | 0.655 | 0.595 |
|  | (3.067) | (3.203) | (2.880) | (0.794) | (0.794) | (0.794) |
| Missing | 0.971 | 1.009 | 0.926 | 0.158 | 0.211 | 0.106 |
|  | (0.405) | (0.422) | (0.388) | (0.433) | (0.435) | (0.434) |
| **Time passed since last shot (ref. Up to one week)** |  |  |  |  |  |  |
| 1 to 2 weeks | 0.942 | 0.913 | 0.925 | 0.185 | 0.225 | 0.176 |
|  | (0.576) | (0.561) | (0.566) | (0.625) | (0.627) | (0.625) |
| 2 weeks to 1 month | 1.348 | 1.332 | 1.312 | -0.208 | -0.172 | -0.220 |
|  | (0.660) | (0.654) | (0.644) | (0.502) | (0.503) | (0.502) |
| 1 to 2 months | 1.289 | 1.285 | 1.247 | -0.221 | -0.186 | -0.239 |
|  | (0.603) | (0.603) | (0.586) | (0.480) | (0.481) | (0.480) |
| 2 to 3 months | 1.333 | 1.333 | 1.285 | -0.186 | -0.153 | -0.208 |
|  | (0.628) | (0.630) | (0.608) | (0.483) | (0.484) | (0.484) |
| 3 to 6 months | 1.535 | 1.512 | 1.476 | -0.092 | -0.070 | -0.112 |
|  | (0.735) | (0.726) | (0.709) | (0.491) | (0.492) | (0.492) |
| 6 months or more | 1.200 | 1.202 | 1.151 | -0.116 | -0.074 | -0.143 |
|  | (0.596) | (0.600) | (0.575) | (0.510) | (0.511) | (0.511) |
| Missing | 1.058 | 1.097 | 1.088 | 0.720 | 0.844 | 0.919 |
|  | (0.872) | (0.913) | (0.911) | (0.856) | (0.860) | (0.867) |
| Not relevant | 4.222 | 4.409 | 3.905 | 0.464 | 0.519 | 0.436 |
|  | (4.039) | (4.224) | (3.745) | (0.901) | (0.901) | (0.902) |
| **(COVID-19 infection & short-term symptoms) X** (**Average household monthly income)** |  |  |  |  |  |  |
| Infected and had silent/light symptoms & Low-Income |  | 0.765 |  |  | -0.078 |  |
|  |  | (0.272) |  |  | (0.371) |  |
| Infected and had silent/light symptoms & Middle-income |  | 0.968 |  |  | -0.144 |  |
|  |  | (0.322) |  |  | (0.343) |  |
| Infected and had silent/light symptoms & High-income |  | 1.850** |  |  | 0.272 |  |
|  |  | (0.569) |  |  | (0.316) |  |
| Infected and had moderate/severe symptoms & Low-income |  | 2.677* |  |  | 1.437*** |  |
|  |  | (1.438) |  |  | (0.479) |  |
| Infected and had moderate/severe symptoms & Middle-income |  | 1.173 |  |  | 0.279 |  |
|  |  | (0.511) |  |  | (0.456) |  |
| Infected and had moderate/severe symptoms & High-income |  | 2.207* |  |  | 0.585 |  |
|  |  | (0.966) |  |  | (0.436) |  |
| **(COVID-19 infection & short-term symptoms) X** (**Ethnicity/religiosity)** |  |  |  |  |  |  |
| Infected and had silent/light symptoms & Non-Ultra-Orthodox Jew |  |  | 0.978 |  |  | 0.043 |
|  |  |  | (0.239) |  |  | (0.253) |
| Infected and had silent/light symptoms & Ultra-Orthodox Jew |  |  | 1.802* |  |  | 0.156 |
|  |  |  | (0.638) |  |  | (0.366) |
| Infected and had silent/light symptoms & Arab Israeli |  |  | 1.004 |  |  | -0.927 |
|  |  |  | (0.606) |  |  | (0.627) |
| Infected and had silent/light symptoms & Other |  |  | 1.000 |  |  | -0.476 |
|  |  |  | (0.000) |  |  | (2.180) |
| Infected and had moderate/severe symptoms & Non-Ultra-Orthodox Jew |  |  | 1.311 |  |  | 0.590* |
|  |  |  | (0.421) |  |  | (0.332) |
| Infected and had moderate/severe symptoms & Ultra-Orthodox Jew |  |  | 5.842*** |  |  | 0.820 |
|  |  |  | (3.606) |  |  | (0.550) |
| Infected and had moderate/severe symptoms & Arab Israeli |  |  | 2.166 |  |  | 1.491*** |
|  |  |  | (1.231) |  |  | (0.549) |
| Infected and had moderate/severe symptoms & Other |  |  | 1.000 |  |  | 0.000 |
|  |  |  | (0.000) |  |  | (0.000) |
| Constant | 0.164* | 0.162* | 0.185* | 0.855 | 0.767 | 0.901 |
|  | (0.161) | (0.159) | (0.181) | (0.925) | (0.927) | (0.927) |
|  |  |  |  |  |  |  |
| Observations | 2,246 | 2,246 | 2,245 | 2,246 | 2,246 | 2,246 |
| Pseudo R2 | 0.0390 | 0.0410 | 0.0410 |  |  |  |
| Adjusted R2 |  |  |  | 0.0490 | 0.0500 | 0.0490 |
| Standard errors in parentheses |  |  |  |  |  |  |
| *** p<0.01, ** p<0.05, * p<0.1 |  |  |  |  |  |  |
| *Note:* columns 1 to 3 show odds ratios of logistic regressions predicting the probability of reporting at least one long-COVID symptom; columns 4 to 6 show estimated coefficients of linear regressions predicting reported long-COVID symptom count. | | | | | | |
