# Supplementary material for: Prevalence of Long-COVID Among Low-Income and Marginalized Groups: Evidence From Israel
Source: Int J Public Health. 2022 Nov 28;67:1605086. doi: 10.3389/ijph.2022.1605086 (PMC9742204; doi:10.3389/ijph.2022.1605086)
Supplement: Supplementary file 2 [file DataSheet1.docx]

# Appendices

## Appendix A1. Itemized long-term symptom experiences, by COVID-19 infection and income (Israel, 2021) *


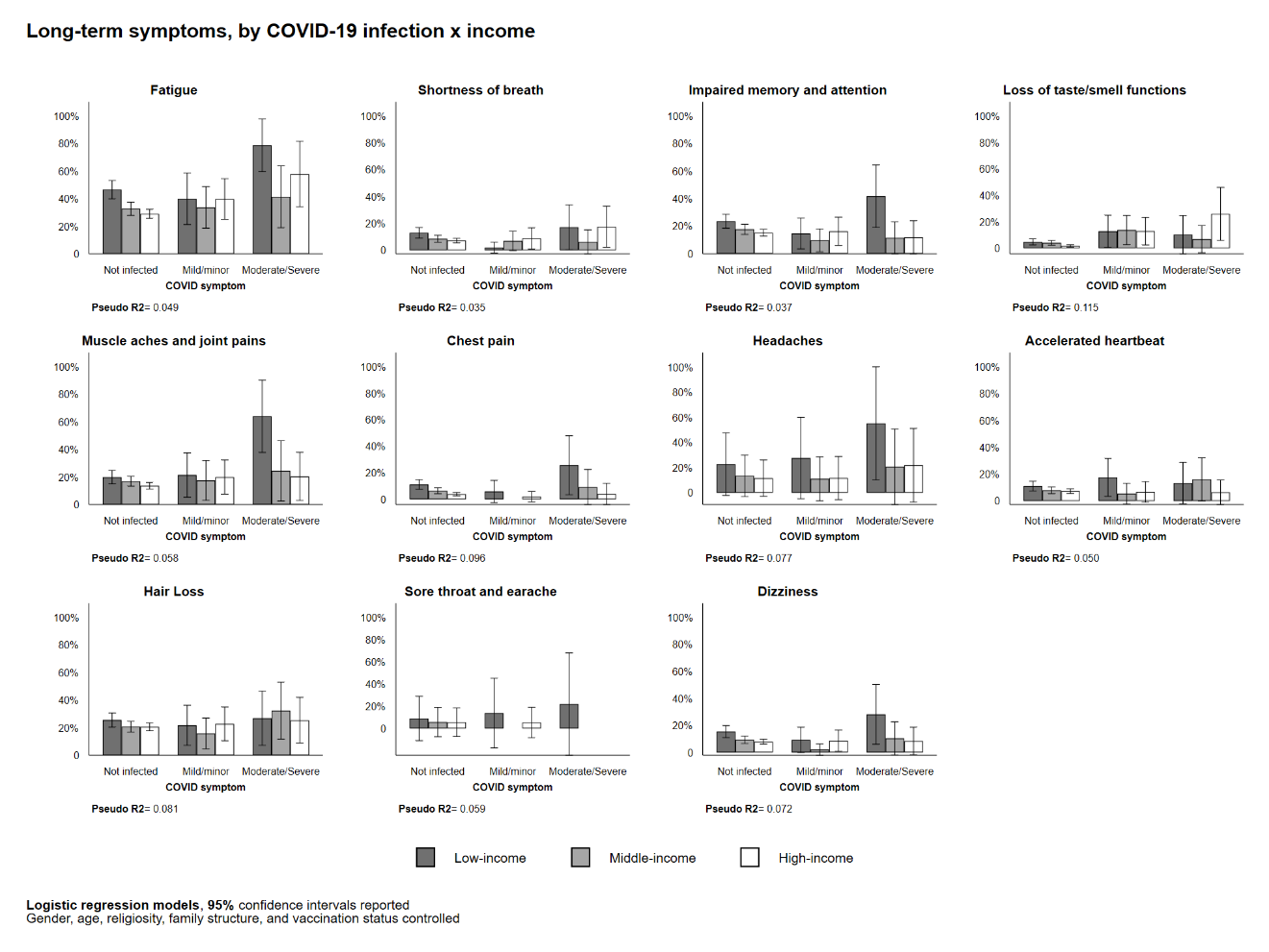


* Marginal probabilities estimated by logistic regressions

## Appendix A2. Itemized long-term symptom experiences by COVID-19 infection and ethnicity/religiosity (Israel, 2021) *


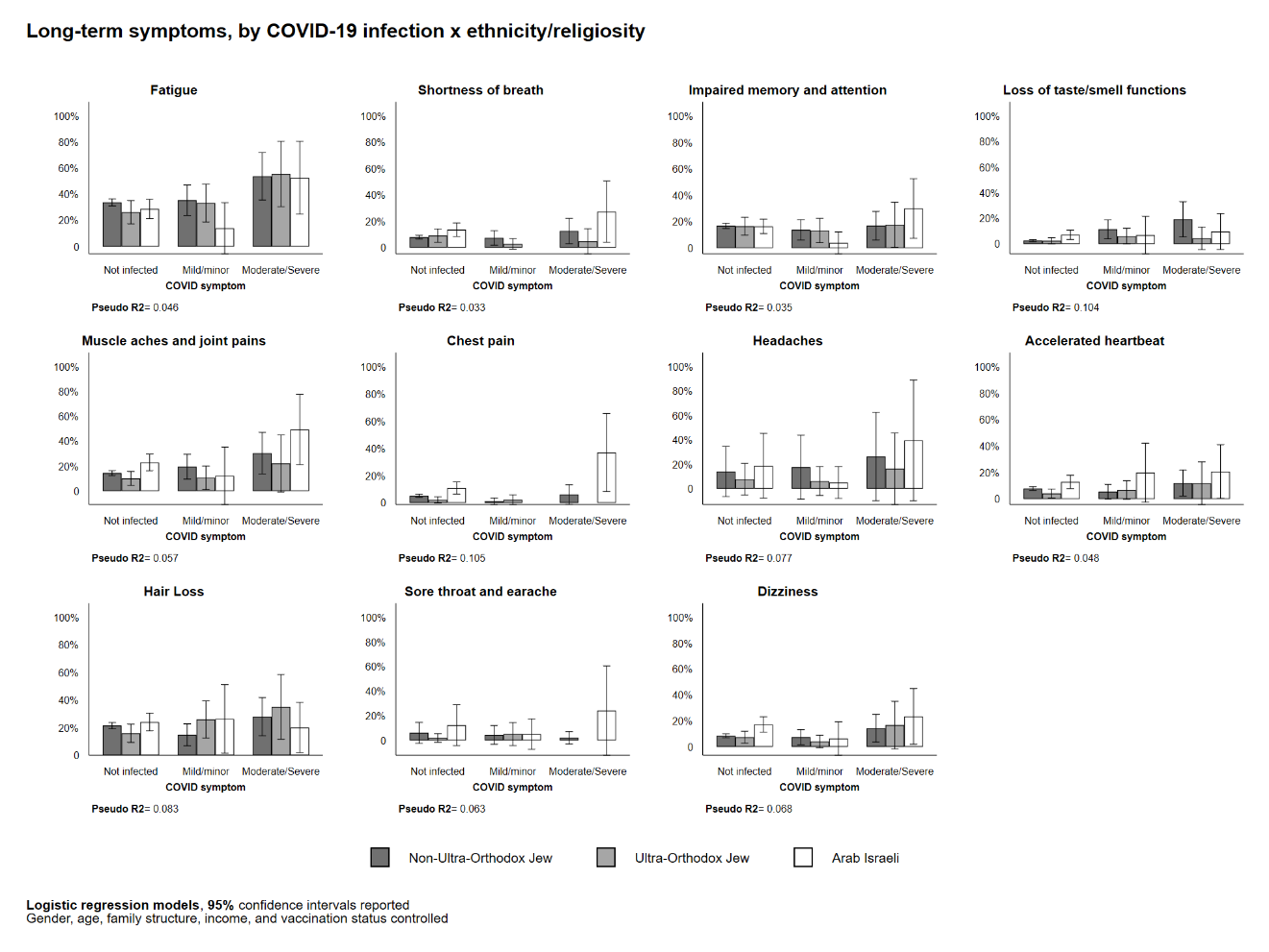


* Marginal probabilities estimated by logistic regressions
